# Sero-prevalence and factors associated with Hepatitis B and C co-infection in pregnant Nigerian women living with HIV Infection

**DOI:** 10.11604/pamj.2014.17.197.2310

**Published:** 2014-03-13

**Authors:** Oliver Chukwujekwu Ezechi, Olufunto Olufela Kalejaiye, Chidinma Vivian Gab-Okafor, David Ayola Oladele, Bamidele Oludare Oke, Zaidat Adesola Musa, Sabdat Ozichu Ekama, Harry Ohwodo, Endurance Agahowa, Titilola Gbajabiamilla, Paschal Mbanefo Ezeobi, Azuka Okwuraiwe, Rosemary Ajuma Audu R, Rosemary Nwakaego Okoye, Agatha Nkiru David, Nkiruka Nonyelum Odunukwe, Dan Ifeanyi Onwujekwe, Innocent Achanya Ujah

**Affiliations:** 1 Clinical Sciences Division, Nigerian Institute of Medical Research, Yaba Lagos Nigeria; 2 Human Virology Laboratory, Nigerian Institute of Medical Research, Yaba Lagos Nigeria; 3 HIV Counseling and Testing Centre, Nigerian Institute of Medical Research, Yaba Lagos Nigeria

**Keywords:** Hepatitis B virus, Hepatitis C virus, HIV, pregnancy

## Abstract

**Introduction:**

Perinatal and horizontal transmission of Hepatitis B occur in areas of high endemicity as most infections are acquired in the first 5 years of life. Unless Hepatitis B and C infected pregnant women identified, and appropriate treatment provided, children born to these women are at high risk of chronic Hepatitis B (and C) virus infection. The objecive of this study was to determined the prevalence and the factors associated with Hepatitis B and C Virus infection in pregnant HIV positive Nigerians.

**Methods:**

A cross sectional study among HIV Positive pregnant women seen at a large PMTCT clinic in Lagos Nigeria. The women were screened for Hepatitis B and C Virus infection at enrollment. HIV viral load, CD4 count, liver transaminases and hemoglobin levels were also determined. Data were managed with SPSS for windows version. Ethical approval was obtained from the Institutions Ethical Review Board.

**Results:**

Of the 2391 studied subjects, 101(4.2%) and 37(1.5%) respectively were seropositive for Hepatitis B and C Virus infection. Twowomen (0. 08%) had triple infections. blood transfusion, (cOR: 2.3; 95% CI:1.1 - 4.6), history of induced abortion (cOR:2. 2;95% CI:1.3 - 3.6), and elevated baseline ALT (cOR:2. 2; 95%CI:2. 2;4.2) were significantly associated with HBV. History of induced abortion was the only factor found to be associated with HIV/ HCV (cOR: 1.9;95%CI:1. 3-3.9).

**Conclusion:**

Hepatitis B Virus infection (4.2%) is relatively common in our environment and associated with induced abortion, blood transfusion and elevated baseline transaminase. Hepatitis C Virus infection (1.5%) is less common and associated with only history of induced abortion.

## Introduction

Globally, hepatitis B virus (HBV) infection is most common form of chronic hepatitis and the leading cause of chronic liver diesase and liver-related deaths [1, 2]. About 350 mllion and 190 million persons are chronically infected with HBV and Heptatitis CVirus (HCV) respectively and are at high risk of death from active hepatitis, cirrhosis and primary hepatocellular carcinoma [3, 4].

The prevalence of HBV infection is greatest in the low income countries of sub-Saharan Africa and South East Asia where 8-10% are chronic carriers and these same regions habours over two-thirds of the global HIV burden [5–7]. In the Western world, chronic HBC and HBV co-infection are found in approximately 30% and 10% of HIV-positive personsrespectively, with only 1% being triply infected with HIV, HBV and HBC [8].

Studies across Nigeria have shown varying prevalences of HBV/HIV co-infection from 9. 2% to as high as 70. 5.% while that of HIV/HCV co-infection ranged between 0. 5% and 14. 7% [9–12]. HBV and HCV infections occur frequently among HIV infected patients because of shared routes of transmission. Most HBV infections have been found to occur within the first 5 years of life in high endemicity regions of Asia and Africa through perinataland horizontal transmission and approximately 25% of infected infants will die of HBV related chronic liver disease in adulthood [13, 14]. Consequently, unless HBV infected pregnant women and associated factors for infection are identified, and adequate treatment provided, their babies are at high risk of HBV infection and its complications later in life.

For both hepatitis HBV and HCV, co-infection with HIV is associated with accelerated progression to cirrhosis and thus a higher mortality. Whether or not HCV directly impacts HIV disease progression remains controversial [15, 16]. Furthermore, individuals co-infected with Hepatitis B and C are risk of hepatoxicity associated with the use of antiretroviral drugs [13–19].

Unfortunately, few studies have addressed co infection of HBV and/or HCV in HIV infected pregnant women more so in Nigeria. This study was conducted to determine the prevalence of and factors associated with HBV and HCV infection among HIV-infected pregnant women in a large HIV treatment centre in southern western Nigerian cosmopolitan city of Lagos.

## Methods

### Study design and Population

The study was a cross-sectional survey conducted at the HIV treatment centre, Nigerian Institute of Medical Research, Lagos. NIMR is the apex medical research Institution in Nigeria charged with the responsibility to conduct research into disease of public health importance in the country. However following the initiation of the Federal Government of Nigeria antiretroviral drug access programme in 2002, it was selected as one of the 25 treatment centres. It was selected principally to provide the research backup for the National HIV programme. Currently the centre provides free comprehensive HIV care, treatment and support for over 19, 000 patients.

Patients are enrolled into the HIV treatment programme following a referral from the HIV Counseling and Testing Centre (HCT), Nigerian Institute of Medical Research Lagos or transfer from other government HIV treatment centres. HIV positive pregnant women on their first visit to the PMTCT clinic between January 2006 and December 2011 who signed an informed consent to participate in the study were recruited for the study. Excluded from the study were women who declined consent to be part of the study, however they were provided PMTCT services.

### Laboratory Tests

Patients are enrolled into the HIV treatment programme following an HIV status determination at the HCT centre as stipulated by the Nigerian National HIV counseling and Testing Guidelines. The HIV status of the pregnant women screened positive are further confirmed at the Human Virology Laboratory (HVL) with Western blot. The women found positive were further evaluated for Hepatitis B and C infection, full blood count, blood chemistry, CD4 count and HIV viral load, using standard methods at the HVL. Hepatitis B Virus or Hepatitis C - infection were based on seropositivity to Hepatitis B surface antigen or Hepatitis C antibody respectively.

### Data Management

At enrollment, information about sociodemographic characteristics, likely mode of HIV acquisition, sexual history, history of blood transfusion, obstetric history, medical history and clinical findings were collected in an antenatal initial case record form specifically designed for the programme. The obtained historical, clinical and laboratory information were thereafter entered into an electronic PMTCT data base by trained data entry clerks using file maker pro. Relevant information for this specific analysis were extracted from the PMTCT Data base and exported to SPSS version 19 for analysis. Frequency distributions were generated and univariate analysis using relevant statistics was performed to identify factors associated with HBV and HCV/HIV coinfection. Logistic regression was used to identify independent determinants for HBV and HCV/HIV coinfection in pregnancy while controlling for potential confounders. The variable with the strongest association in the univariate model was estimated first, followed by others in descending other. In the analysis, the comparison group was HIV positive pregnant women without HBV and HCV coinfection. P<0. 05 was considered to be statistically significant.

### Ethical Issues

Approval for the study was obtained from the Institutional Review Board of the Nigerian Institute of Medical Research, Lagos Nigeria. Written informed consents were obtained from participants for the use of their data for the study however women who declined consent to participate in study were provided care like other women according to Nigerian National PMTCT and HIV treatment guidelines but excluded from research.

## Results

A total number of 2392 HIV positive pregnant women enrolled in the PMTCT programme during the study period were invited to participate in the study. All but one signed a written informed consent to participate in the study. Information of the 2391 women were used for this analysis.

**Characteristics of the women in the study:** the sociodemographic, reproductive and laboratory characteristics of the 2391 HIV positive women studied is shown inTable 1. The mean age of the women was 29.5 ± 4.4 years with range 14 - 44 years. Majority of the women were within the age group 20-29 (51.9%). Most of the women had at least a previous delivery (71.9%), were married (81.5%), had at least a secondary education (81.7%) and were gainfully employed (78.1%). The predominant route of HIV infection was through heterosexual contact (78.5%). The mean gestational age at enrollment was 23. 7±11.7 years (range 4-41), with majority (85.5%) of the women enrolling after their first pregnancies.

**Table 1:** Sociodemographic, reproductive and biologic characteristics of the 2391 pregnant HIV positive women studied (2006 -2011)

Characteristics	Number of respondents (%)
**Age (Years)**	
Less than 20	18(0.8)
20 – 29	1224(51.9)
30 – 39	1109(46.4)
40 – 49	40(1.7)
**Marital Status**	
Single	352(14.7)
Married	1948(81.5)
Separated/Divorced	34(1.4)
Widow	57(2.4)
**Educational level completed**	
None	71(3.0)
Primary	365(15.3)
Secondary	1155(48.3)
Tertiary	800(33.4)
**Occupation**	
Student	274(11.5)
Unemployed/House wife	248 (10.4)
Trading	675(28.2)
Unskilled	347(14.5)
Artisan	361(15.1)
Civil servant	348(14.6)
Professional/Business Executive	138(5.8)
**Mode of acquisition of HIV**	
Heterosexual Contact	1876(78.5)
Blood and Blood product	129(5.4)
MTCT	2(0.1)
Unknown	386(16.1)
**History of Induced abortion**	
Yes	1506(63.0)
No	885(37.0)
**Parity**	
0	671(28.1)
1 - 2	892(37.3)
3 – 4	595(24.9)
5 and above	233(9.7)
**Gestational age**	
First trimester	347(14.5)
2nd Trimester	1041(43.5)
3rd Trimester	1003(42.0)
**CD4 cell count**	
<200	811(33.9)
201-349	695(29.1)
350 – 500	454(19.0)
>500	431(18.0)
**Viral Load**	
0 – 1000	410(17.1)
1,001 – 10,000	611(25.6)
10,001 – 100,000	835(34.9)
>100,000	535(22.4)
**Alanine transaminases (ALT)**	
≤45	2246(93.9)
>45	145(6.1)

**Prevalence of and associated factors for HBV co-infection:** of the 2391 women in the study, 101 were found to be Hepatitis B surface antigen positive; HBsAg prevalence of 4. 2% (95% CI 3. 05-5. 9%). Prevalence ranged from 3. 3% (95% CI 2. 1-7. 3%; 14 of 429) for women aged less than 20 years to 4.5% (95% CI 2.4 - 9.1%; 76 of 1687) for women aged between 20-35 years. Table 2 shows the association between select maternal characteristics and Hepatitis B co-infection. Although a history of blood transfusion (cOR:2.3; 95% CI:1.1 - 4.6), history of induced abortion (cOR:2. 2;95% CI:1.3 - 3.6), viral load greater than 100, 000 copies (cOR:1. 9;95%CI:1.2 - 3.2) and ALT ≥45 (cOR: 2.2; 95% CI: 2.2 - 4.2) were found to be associated with Hepatitis B co-infection at univariate analysis. However after adjustment at multivariate analysis while controlling for possible confounders showed that history of blood transfusion (aOR: 2.4; 95% C:1.4 - 4.4), induced abortion (aOR: 1.4; 95% CI: 1.4 - 3.1)and elevated baseline ALT (aOR: 2.5; 95% CI:1.3 - 3.5) retained their independent association with Hepatitis B co-infection.

**Table 2:** Association between patient's characteristics and Hepatitis B co-infection among HIV positive pregnant women

Characteristics	HIV positive pregnant women		P value	Crude OR (95% CI)	Adjusted OR (95% CI)
	HBV Positive (%) N=101	HBV negative (%) N=2290			
**Age (years)**					
<20	11(10.9)	264(11.5)	0.83	0.90(0.44 – 1.74)	0.94(0.49 – 1.79)
20 – 35	76(75.2)	1611(70.3)		1.0(Ref)	1.0(Ref)
>35	14(13.9)	415(18.1)	0.32	0.72(0.38 - 1.31)	0.79(0.41 - 1.73)
**Parity**					
<2	65(64.4)	1498(65.4)	0.9	1.0(Ref)	1.0(Ref)
≥2	36(35.6)	792(34.6)		1.1(0.68 – 1.62)	1.4(0.81 – 1.99)
**Marital Status**					
Married	87(86.1)	1861(81.3)	0.27	1.0(Ref)	1.0(Ref)
Not married	14(13.9)	429(18.7)		0.7(0.38 – 1.27	0.9(0.51 – 1.61)
**Educational level completed**					
<secondary	21(20.8)	415(18.1)	0.58	1.2(0.70 – 1.99)	1.2(0.74 – 1.59)
≥Secondary	80(79.2)	1875(81.9)		1.0(Ref)	1.0(Ref)
Work Status					
Working	72(71.3)	1797(78.5)	0.11	1.0(Ref)	1.0(Ref)
Not working	29(28.7)	493(21.5)		1.5(0.92 – 2..33)	1.6(0.97 – 2.03)
**Mode of acquisition of HIV**					
Heterosexual Contact	90(89.1)	1808(96.0)	0.02	1.0(Ref)	1.0(Ref)
Blood and Blood product	11(10.9)	96(4.0)		2.3(1.12 – 4.61)	2.4(1.35 – 4.40)^a^
**History of Induced abortion**					
Yes	79(78.2)	1427(62.3)	0.002	2.17(1.31 – 3.62)	2.2(1.4 – 3.1) b
No	22(21.8)	863(37.7)		1.0(Ref)	1.0(Ref)
**HIV Viral Load**					
<10,000	29(28.7)	992(43.3)	0.23	0.71(0.42 – 1.21)	0.9(0.78 – 1.01)
10,001 – 100,000	33(32.7)	802(35.0)		1.0(Ref)	1.0(Ref)
>100,000	39(38.6)	496(21.7)	0.01	1.91(1.16 – 3.16)	1.5(0.99 – 4.06)
**CD4 cell count**					
<350	64(63.4)	1442(63.0)	0.89	1.08(0.69 – 1.90)	1.1(0.81 – 1.99)
350 – 500	18(17.8)	436(37.0)		1.0(Ref)	1.0(Ref)
>500	19(18.8)	412	0.87	1.12(0.55 - 2.26)	1.2(0.75 - 2.16)
**Alanine Transaminases**					
<45	89(88.1)	2157(94.2)	0.02	1.0(Ref)	1.0(Ref)
≥45	12(11.9)	133(5.8)		2.19(1.11 – 4.23)	2.5(1.30 – 3.51) c

a Note: Adjusted for age, parity and marital status

b Adjusted for age, parity and marital status and educational status

c Adjusted for age, CD4 count and route of infection

**Prevalence of and associated factors for HCV co-infection:** of the 2391 women in the study, 37 were found to be Hepatitis C antibody positive; HCV antibody prevalence of 1. 5% (95% CI 1. 23 - 3. 1%). It ranged between 1. 4% (95% CI 1. 1-6. 4%; 32 of 2225) for women aged between 20 and 35 years and 11. 1% (95% CI 4.5 - 15.1%; 2 of 18) for women aged less than 20 years. The association between select maternal characteristics and Hepatitis C co-infection is shown in Table 3. Univariate and multivariate analysis after controlling for confounders showed that only a history of induced abortion was found to be associated with Hepatitis C co-infection (aOR:1. 9;95% CI:1. 3- 3. 9).

**Table 3:** Association between patient's characteristics and Hepatitis C co-infection among HIV positive pregnant women

Characteristics	HIV positive pregnant women		P value	Crude OR (95% CI)	Adjusted OR (95% CI)
	HBC Positive (%) N=37	HBC negative (%) N=2354			
**Age (years)**					
<20	2(5.4)	16(0.68)	0.74	0.57(0.09 – 1.74)	0.69(0.32 – 4.77)
20 – 35	32(86.5)	2193(93.2)		1.0(Ref)	1.0(Ref)
>35	3(8.1)	145(6.2)	0.48	1.42(0.34 – 4.9)	0.93(0.41 - 6.73)
**Mode of acquisition of HIV**					
Heterosexual Contact	33(89.2)	1867(79.5)		1.0(Ref)	1.0(Ref)
Blood and Blood product	4(10.8)	103(4.0)	0.13	2.20(0.65 – 6.66)	1.7(0.45 – 9.40)
**History of Induced abortion**					
Yes	31(83.8)	1471(62.5)	0.01	3.09(1.22 – 8.27)	1.9(1.3 – 3.9) a
No	6(16.2)	879(37.5)		1.0(Ref)	1.0(Ref)
**HIV Viral Load**					
<10,000	13(28.7)	1008(43.3)	0.86	1.18(0.47 – 3.01)	1.05(0.65 – 4.32)
10,001 – 100,000	9(32.7)	826(35.0)		1.0(Ref)	1.0(Ref)
>100,000	15(38.6)	520(21.7)	0.03	2.65(1.08 – 6.59)	1.9(0.97 – 5.23)
**CD4 cell count**					
<350	15(40.5)	1491(63.3)	0.89	1.08(0.69 – 1.90)	1.1(0.81 – 1.99)
350 – 500	13(35.1)	441(18.7)		1.0(Ref)	
>500	9(24.3)	422(18.0)	0.87	1.12(0.55 - 2.26)	1.2(0.75 - 2.16)
**Alanine Transaminases**					
<45	32(86.5)	2214(94.1)	0.07	1.0(Ref)	1.0(Ref)
≥45	5(13.5)	140(5.9)		2.47(0.83 – 6.78)	1.9(0.91 – 3.98)

a Note: Adjusted for age, parity and marital status and route of infection and hepatitis B infection

**Triple infection among the women:** two of the 2391 women in the cohort, tested positive to both HBV and HCV infection; HCV/HBV prevalence of 0. 08%.

## Discussion

A few studies across Africa have shown a variation in the prevalence of HIV/HBV co infection in pregnancy. The prevalence of HBV /HIV co-infection in pregnant women of 4. 2% in our study was similar to 4. 2% by Eke and colleagues in Nnewi, South East Nigeria and 4. 1% by Pirrillo and colleagues from Rwanda, South Africa [20, 21]. Landes and colleagues in the European Collaborative studyalso reported a similar prevalence of 4.9% [22]. A likely explanation for this is that they found that HbsAg positivity was associated with black African origin of whom accounted for one fifth of their study population [22]. Higher prevalence of 8. 9 and 9. 0% respectively were reported by Adesina et al. from Ibadan Nigeria and Rouet et al. from Abidjan, Cote d'Ivoire [23, 24]. The differences in social and cultural practices as well as varying sample size, test kit sensitivity and specificity may have been responsible and accounted for the variation in prevalence rates in the Nigerian studies [25]. However, a lower prevalence of 1. 5% was reported by Santiago-munozet al from Texas, USA which can be explained by North America being a low endemicity area for Hepatitis B [26].

The factors associated with HBV co-infection in our cohort were a history of blood transfusion, (aOR:2. 4; 95% CI:1.4 - 4.4), induced abortions(aOR:2. 2;95% CI:1.4 - 3.9), and elevated baseline ALT levels (aOR:2. 5; 95% CI:1.3-3.5). Potential causes for adult transmission for Hepatitis B (and HIV) exist including blood transfusion. It is likely that these sources of blood were not screened for Hepatitis B [27]. The association of HBV/HIV co infection and induced abortions may be related to the fact that abortion is not legal in Nigeria and a majority of womenwith unwanted pregnancy patronize quacks who often perform under unhygienic environment using unsterilized equipment. Also unwanted pregnancy may be a surrogate for unprotected sexual intercourse which is a known risk factor for both hepatitis B and HIV infections.

Co-infection with HIV has been found to worsen Hepatitis B (and C) infection and progression [15, 16]. However, the effect of HBV and HCV directly on HIV disease progression remains controversial [15, 16]. Our study, similar to others, showed no association between HIV disease severity by CD4 count and HIV viral load with HBV co infection [22, 23].

Elevated Transaminase levels were associated with HIV/HBV co-infection in cohort which was similarly reported by studies from Ibadan [27]. A number of causes of elevated liver enzyme transaminases in Hepatitis B infection have been identified in other studies and include HBeAg seroconversion, acute infection with hepatitis D and uninhibited HBV viral replication [13]. These causes however were not evaluated in our study. Studies from China have linked ALT levels of at least >0. 5-2 times the upper limit of normal in patients with Hepatitis B infection with a greater risk of development of complications of chronic hepatitis [28]. It may suffice to add that, co-infection with Hepatitis B increases the risk of hepatotoxicity from antiretroviral therapy at least 3-5 fold and can pose challenges to treatment modalities [19, 29].

Our study did not find an association between maternal age and HIV/HBV co-infection. This finding differed from studies in Europe and South -Eastern Nigeria where women aged between 25-29 years and 20-30 years respectively were found to be at a significant risk of HIV/HBV co- infection [22, 25]. The Nigerian researchers attributed this to the higher prevalence of HIV as well as the likelihood of high sexual activity in these age groups [25].

The 1. 5% prevalence of HCV/HIV co-infection in this study compares with 1.5% by Adesina et al from Ibadan, 1. 0% by Rouetet al from Abidjan and 1.3% by Simporeet al from Ouagadougou, Burkina Faso [23, 24, 29]. Higher prevalence of 4. 9% and 12. 3% in Texas, USA, and Europe were reported by Santiago-munozet al and Landeset al from Europe and was attributed to intravenous drug use in these populations [22, 26]. At present intravenous drug use is not a major challenge in our environment and thus may have accounted for the lower prevalence.

The only factor found to be associated with HCV/HIV co-infection in our cohort was a history of induced abortion. (aOR:1. 9;95% CI:1. 3- 3. 9). Hepatitis C appears to be of a lower prevalence than Hepatitis B in our setting but does share similar modes of transmission as Hepatitis B and HIV. Maternal age, HIV viral load and CD4 counts were not associated with HCV/HIV co infection in this study. This however differed from findings from the European collaborative study in which maternal age>35years and high HIV viral levels were associated with HCV/HIV co-infection [22]. Although, the effect of HBV and HCVinfection on HIV disease progression remains controversial, it has been suggested that, co-infection between Hepatitis C virus and HIV may be associated with a rapid decline in the CD4 count, rapid progression of HIV infection and increased morbidity and mortality [30].

A very small proportion of our cohort(0. 08%) had triple infections of HBV, HCV and HIV which is similar to findings of 0. 1% at Ibadan suggesting that all 3 co-infections in pregnant women is uncommon in our setting [23]. Although our study did try to compare as best as possible with other previous studies, we did have our limitations. As we were only able to look at HBsAg positivity and Hepatitis C antibody as markers for HBV and HCV respectively, and had no information on other serum markers, we were therefore unable to investigate serological evidence of active or past infection for either HBV of HCV or prior immunization for HBV.

## Conclusion

In view of the relatively high prevalence of 4.2% for HBV and 1.5% for HCV in this study, routine screening of all pregnant women for HBV and HCV as well as prompt referral and treatment to reduce perinatal transmission and thus chronic hepatitis in children should be adopted as standard of care. However in rural areas with limited capacity, a high index of suspicion should be entertained in pregnant women with HIV infection, previous blood transfusion and a history of induced abortion. This category of women should be referred to facility with HBV and HCV screening.
